# Supplementary material for: Co-stimulation with opposing macrophage polarization cues leads to orthogonal secretion programs in individual cells
Source: Nat Commun. 2021 Jan 12;12:301. doi: 10.1038/s41467-020-20540-2 (PMC7804107; doi:10.1038/s41467-020-20540-2)
Supplement: Supplementary file 1 — Supplementary Information [file 41467_2020_20540_MOESM1_ESM.pdf]

# **Co-stimulation with opposing macrophage polarization cues leads to orthogonal secretion programs in individual cells**

Muñoz-Rojas *et al.*

Supplementary Information

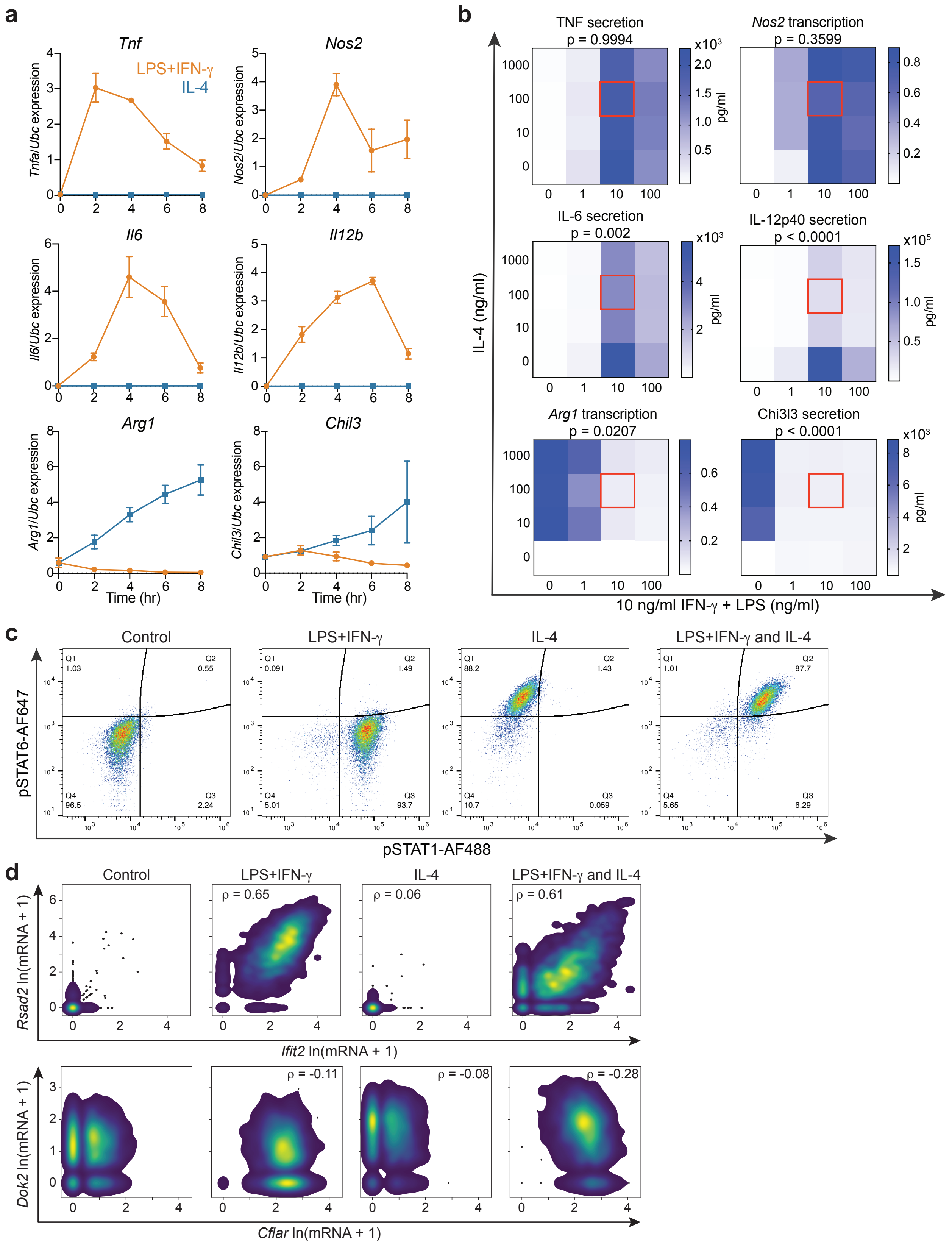


**Supplementary Fig. 1 (associated with Fig. 1). Timing and dose dependence of cross-inhibition between LPS+IFN-γ and IL-4 responses in BMDMs. a** Time course of transcription in BMDMs after stimulation with 10 ng/ml LPS+10 ng/ml IFN-γ or 100 ng/ml IL-4 for selected targets measured by RT-qPCR (mean ± SEM, n=3 biological replicates). **b** Heatmaps of response in BMDMs after stimulation for 24 h with 10 ng/ml IFN-γ + a range of LPS doses combined with a range of doses of IL-4. Data represent *Arg1* and *Nos2* transcript levels measured by RT-qPCR and Chi3l3, IL-6, IL-12p40, and TNF protein levels measured by ELISA (mean, n=3 biological replicates). *P*-value represents the significance of the interaction between the stimulations calculated by a 2-way ANOVA. **c** Phospho-Stat1 and phospho-Stat6 expression by flow cytometry in BMDMs after stimulation with 10 ng/ml LPS+10 ng/ml IFN-γ, 100 ng/ml IL-4, or the combination for 30 min. **d** Density scatter plots of scRNA-seq transcript counts across individual cells for *Rsad2* vs. *Ifit2* (top) and *Dok2* vs. *Cflar* (bottom). ρ indicates Spearman correlation coefficient. Gene expression is shown as ln(transcript count +1). Source data are provided as a Source Data file.

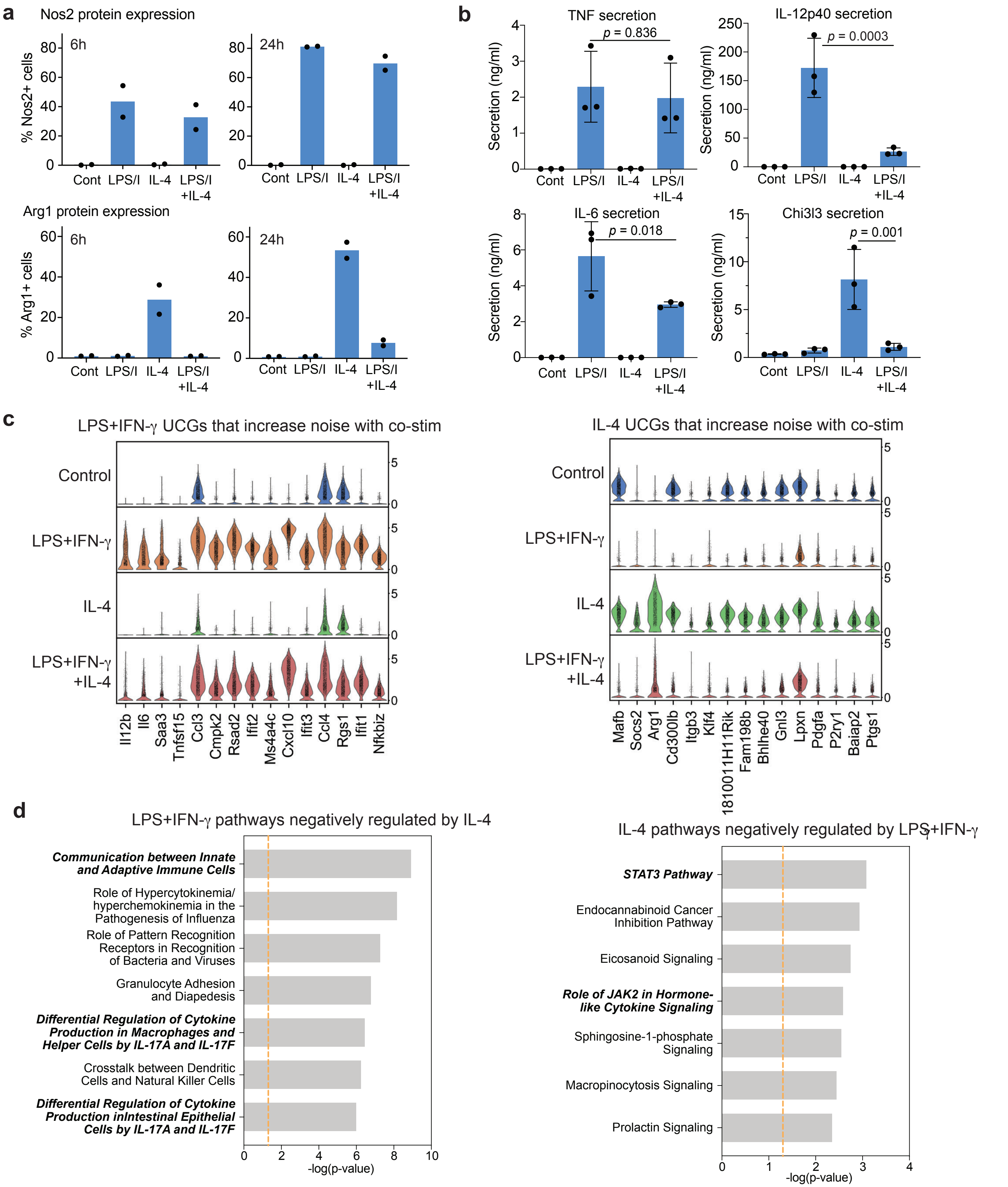


**Supplementary Fig. 2 (associated with Fig. 2). Cross-inhibition and cell-to-cell noise in gene expression after co-stimulation.** **a** Levels of Nos2 (top) and Arg1 (bottom) protein detected by flow cytometry after stimulation for 6 h (left) and 24 h (right) with 10 ng/ml LPS+10 ng/ml IFN-γ, 100 ng/ml IL-4, or the combination (mean, n=2 biological replicates). **b** Levels of TNF, IL-12p40, IL-6 and Chi3l3 secretion after stimulation for 24 h with 10 ng/ml LPS+10 ng/ml IFN-γ, 100 ng/ml IL-4, or the combination (mean ± SEM, n=3 biological replicates). P-values determined with two-sided one-way ANOVA with Sidak correction for multiple comparisons. **c** Violin plots of scRNA-seq measurements of LPS+IFN-γ-induced (top) and IL-4-induced (bottom) genes with the highest change in cell-to-cell gene expression noise measured by Fano factor. **d** Result from Ingenuity Pathway Analysis listing the top 7 canonical pathways that are associated with the set of LPS+IFN-γ-induced (top) or IL-4-induced (bottom) genes that are inhibited after co-stimulation. *P*-values indicate the significance of the overlap between the canonical pathway and the inhibited genes (determined by one-sided Fisher's exact test). Source data are provided as a Source Data file.

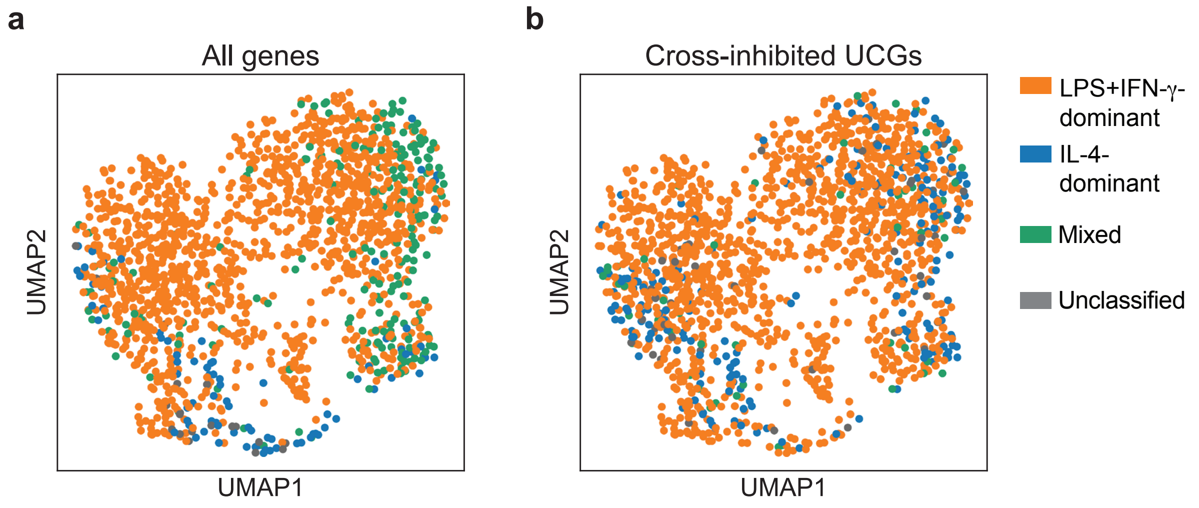


**Supplementary Fig. 3 (associated with Fig 3). Neural network classifier results identify clusters of mixed and single-stimuli-dominant cells. a-b** UMAP representation of classification results from the neural network classifier trained on all genes (**a**) or UCGs that displayed cross-inhibition after co-stimulation (**b**).

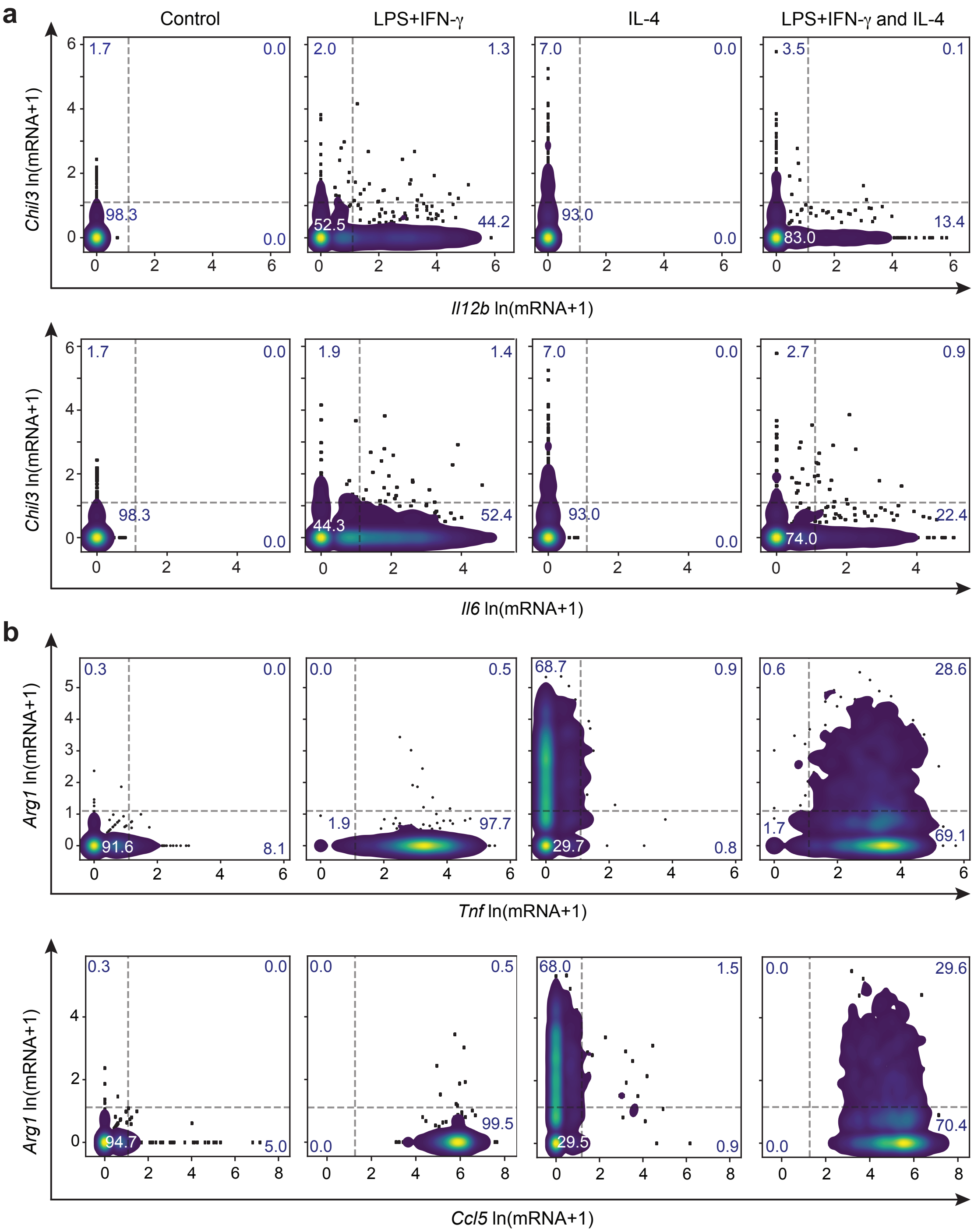


**Supplementary Fig. 4 (associated with Fig. 4). Orthogonal expression is observed for *Il6* and *Il12b* with *Chil3* but not for *Tnf* and *Ccl5* with *Arg1*.** Density scatter plots of scRNA-seq transcript counts across individual cells for *Chil3* vs. *Il12b* (**a**, top) and *Il6* (**a**, bottom); and for *Arg1* vs. *Tnf* (**b**, top) and *Ccl5* (**b**, bottom). Data is represented as ln(transcript count +1).

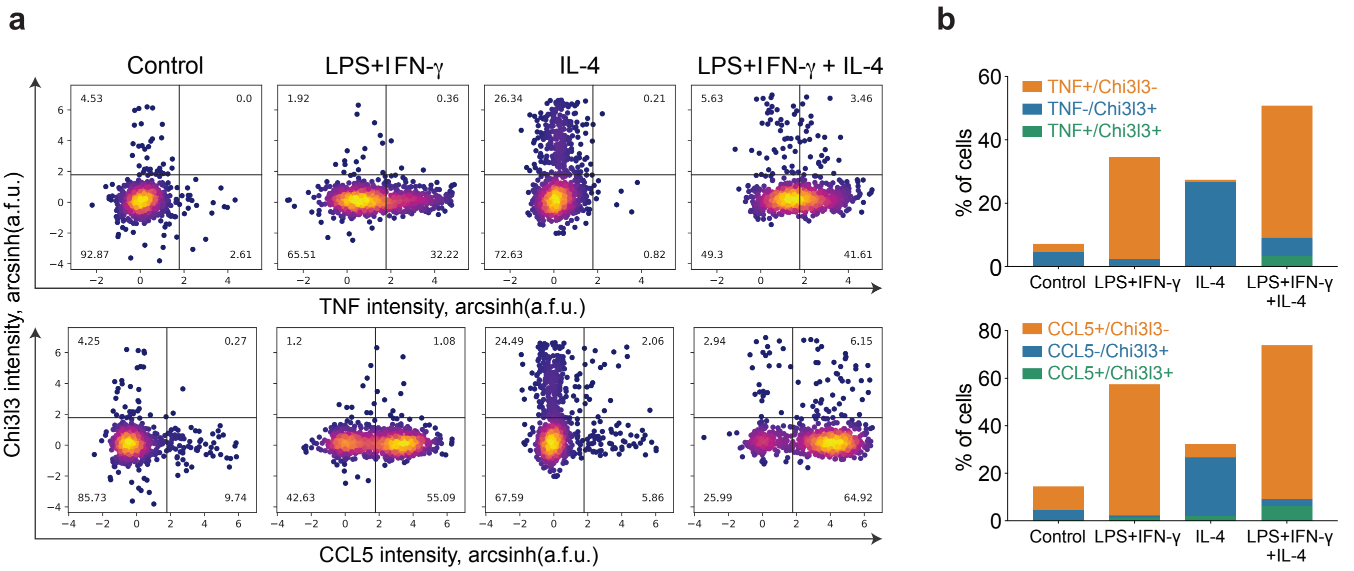


**Supplementary Fig. 5 (associated with Fig. 5). TNF and CCL5 are not orthogonally secreted with Chi3l3.** A multiplexed single-cell secretion assay was used to measure cytokine/chemokine production in individual BMDMs stimulated for 48 h with media alone, 10 ng/ml LPS+10ng/ml IFN-γ, 100 ng/ml IL-4, or both. **a** Density scatter plots of single-cell secretion intensity (a.f.u.) across individual cells for Chi3l3 vs TNF (top) and Chi3l3 vs CCL5 (bottom). **b** Quantification of single-positive and double-positive cells after 48-hour co-stimulation for secretion of Chi3l3 and TNF (top) and Chi3l3 and CCL5 (bottom). Data is pooled from 2 biological replicates.

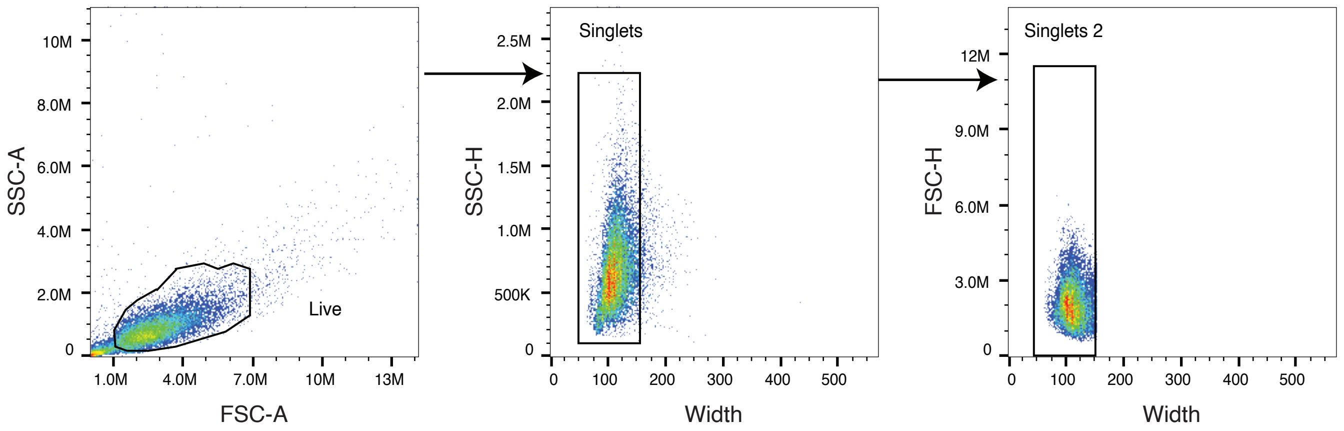


**Supplementary Fig. 6. Gating strategy for flow cytometry experiments.** Gating strategy for gating of live, singlet BMDMs.

**Supplementary Table 1. Capture and detection ELISA antibody pairs for secretion profiling.**

| Antibody Pair | Vendor | Catalog No. |
|---|---|---|
| TNF | eBioscience | 88-7324-88 |
| CCL17 | R&D | DY529 |
| IL-12p40 | BD Biosciences | 555165 |
| IL-10 | BD Biosciences | 555252 |
| MMP9 | R&D | DY6718 |
| IL-6 | R&D | DY406 |
| IGF-I | R&D | DY791 |
| CCL2 | R&D | DY479 |
| CCL8 | R&D | DY790 |
| Chi3l3 | R&D | DY2446 |
| CCL3 | R&D | DY450 |
| CXCL1 | R&D | DY453 |
| GM-CSF | R&D | DY415 |
| CCL22 | R&D | DY439 |
| CCL5 | R&D | DY478 |

**Supplementary Table 2. Primers used for qPCR.**

| Target | Forward Primer (5' to 3') | Reverse Primer (5' to 3') |
|---|---|---|
| *Nos2* | GTTCTCAGCCCAACAATACAAGA | GTGGACGGGTCGATGTCAC |
| *Arg1* | CTCCAAGCCAAAGTCCTTAGAG | AGGAGCTGTCATTAGGGACATC |
| *Klf4* | GACTAACCGTTGGCGTGAGG | GTCTAGGTCCAGGAGGTCGT |
| *Nfkbiz* | GAGTCCCGTCCCAGAGGTG | ACTCTGTGTCTTAAACTCATCCAC |
| *Il6* | TCCTCTCTGCAAGAGACTTCC | TTGTGAAGTAGGGAAGGCCG |
| *Tnf* | CCCTCACACTCAGATCATCTTCT | GCTACGACGTGGGCTACAG |
| *Il12b* | CGCCACACAAATGGATGCAA | TGTGTCCTGAGGTAGCCGTA |
| *Chil3* | CAGGTCTGGCAATTCTTCTGAA | GTCTTGCTCATGTGTGTAAGTGA |
| *Ubc* | ACCACCAAGAAGGTCAAACAGG | TAAGACACCTCCCCCATCAC |
