# Supplementary material for: Co-stimulation with opposing macrophage polarization cues leads to orthogonal secretion programs in individual cells
Source: Nat Commun. 2021 Jan 12;12:301. doi: 10.1038/s41467-020-20540-2 (PMC7804107; doi:10.1038/s41467-020-20540-2)
Supplement: Supplementary file 3 — Descriptions of Additional Supplementary Files [file 41467_2020_20540_MOESM3_ESM.pdf]

Descriptions of Additional Supplementary Files

Supplementary Data 1
Description: Associated with Fig. 1, Excel spreadsheet containing the list of upregulated and downregulated genes after stimulation with LPS+IFN-γ or IL-4 compared to unstimulated cells.

Supplementary Data 2
Description: associated with Fig. 5, Excel spreadsheet containing the intensity values of single-cell secretion data of macrophages stimulated for 48 hours with media alone, LPS+IFN-γ, IL-4, or both.
