# Supplementary material for: Co-stimulation with opposing macrophage polarization cues leads to orthogonal secretion programs in individual cells
Source: Nat Commun. 2021 Jan 12;12:301. doi: 10.1038/s41467-020-20540-2 (PMC7804107; doi:10.1038/s41467-020-20540-2)
Supplement: Supplementary file 6 — Reporting Summary [file 41467_2020_20540_MOESM6_ESM.pdf]

nature research

Corresponding author(s): Kathryn Miller-Jensen

Last updated by author(s): Oct 30, 2020

Reporting Summary

Nature Research wishes to improve the reproducibility of the work that we publish. This form provides structure for consistency and transparency in reporting. For further information on Nature Research policies, see our Editorial Policies and the Editorial Policy Checklist.

Statistics

For all statistical analyses, confirm that the following items are present in the figure legend, table legend, main text, or Methods section.

n/a	Confirmed	
☐	☒	The exact sample size (n) for each experimental group/condition, given as a discrete number and unit of measurement
☐	☒	A statement on whether measurements were taken from distinct samples or whether the same sample was measured repeatedly
☐	☒	The statistical test(s) used AND whether they are one- or two-sided<br>*Only common tests should be described solely by name; describe more complex techniques in the Methods section.*
☒	☐	A description of all covariates tested
☐	☒	A description of any assumptions or corrections, such as tests of normality and adjustment for multiple comparisons
☐	☒	A full description of the statistical parameters including central tendency (e.g. means) or other basic estimates (e.g. regression coefficient) AND variation (e.g. standard deviation) or associated estimates of uncertainty (e.g. confidence intervals)
☐	☒	For null hypothesis testing, the test statistic (e.g. F, t, r) with confidence intervals, effect sizes, degrees of freedom and P value noted<br>*Give P values as exact values whenever suitable.*
☒	☐	For Bayesian analysis, information on the choice of priors and Markov chain Monte Carlo settings
☒	☐	For hierarchical and complex designs, identification of the appropriate level for tests and full reporting of outcomes
☐	☒	Estimates of effect sizes (e.g. Cohen's d, Pearson's r), indicating how they were calculated

Our web collection on statistics for biologists contains articles on many of the points above.

Software and code

Policy information about availability of computer code

Data collection	Single-cell secretion data was acquired using custom code written in MATLAB (vR2017a) available at: https://github.com/Miller-JensenLab/Single-Cell-Analysis.
Data analysis	10X single-cell RNA raw sequencing data were processed using Cell Ranger software (v2.1.1). Further analysis was done using custom python scripts. The custom code used to analyze the data in the paper and generate the figures is available at the following GitHub repository: [https://github.com/Miller-JensenLab/munoz-rojas_NatCommunications2020]. This analysis was done in python v3.7.6, using the following packages: scanpy v1.5.1, anndata2ri v1.0.2, rpy2 v3.2.4, pandas v1.0.3, seaborn v0.10.1, scikit-learn v0.22.1, scipy v1.4.1, and openensembles v1.1.1, as well as R package scran v1.12.1.<br><br>Single-cell secretion data was analyzed using custom code written in MATLAB (vR2017a) available at: [https://github.com/Miller-JensenLab/Single-Cell-Analysis], as well as custom python scripts.<br><br>Flow cytometry analysis was performed with Flowjo (v10.7.1), plotting and statistics were performed with Graphpad Prism (v8.4.3)

For manuscripts utilizing custom algorithms or software that are central to the research but not yet described in published literature, software must be made available to editors and reviewers. We strongly encourage code deposition in a community repository (e.g. GitHub). See the Nature Research guidelines for submitting code & software for further information.

Data

Policy information about availability of data

All manuscripts must include a data availability statement. This statement should provide the following information, where applicable:

- Accession codes, unique identifiers, or web links for publicly available datasets
- A list of figures that have associated raw data
- A description of any restrictions on data availability

Raw and processed scRNA-seq data that support the findings of this study are deposited in the National Center for Biotechnology Information Gene Expression Omnibus (GEO), accession code: GSE161125 [https://www.ncbi.nlm.nih.gov/geo/query/acc.cgi?acc=GSE161125]. All other data supporting the findings of this study are available with the article and its supplementary information files, and from the corresponding author upon reasonable request. Source data are provided with this paper.

Field-specific reporting

Please select the one below that is the best fit for your research. If you are not sure, read the appropriate sections before making your selection.

[x] Life sciences [ ] Behavioural & social sciences [ ] Ecological, evolutionary & environmental sciences

For a reference copy of the document with all sections, see nature.com/documents/nr-reporting-summary-flat.pdf

Life sciences study design

All studies must disclose on these points even when the disclosure is negative.

Sample size	All experiments were performed with bone-marrow-derived macrophages (BMDMs) isolated from the bone marrow of C57BL/6 mice and frozen in aliquots such that they are comparable to standard in vitro cell culture experiments. For comparing population means of in vitro cell culture experiments, a sample size of n=3 is generally accepted as sufficient to obtain standard deviations with significance set at $p < 0.05$. Therefore, we used a sample size of n=3.
Data exclusions	We used pre-established criteria in the single-cell analysis field to exclude low quality cells as follows: dying cells were identified as those cells with a high proportion of mitochondrial gene transcripts and were excluded. Doublets and empty droplets were also excluded based on their number of genes and UMIs detected.
Replication	Single-cell RNA sequencing experiment was done once due to high cost as standard in the field. Single-cell secretion experiments were repeated in two independent biological replicates and pooled for data analysis. Cell-population experiments in support of single-cell findings were all repeated in three independent biological experiments.
Randomization	As described in "Sample size" all experiments were performed with BMDMs isolated from the bone marrow of C57BL/6 mice and frozen in aliquots such that they are comparable to standard in vitro cell culture experiments. There is no distinction between the starting cell samples used in these in vitro experiments and therefore no randomization was necessary.
Blinding	There is no distinction between the starting cell samples used in these in vitro experiments and therefore no blinding was necessary.

Reporting for specific materials, systems and methods

We require information from authors about some types of materials, experimental systems and methods used in many studies. Here, indicate whether each material, system or method listed is relevant to your study. If you are not sure if a list item applies to your research, read the appropriate section before selecting a response.

Materials & experimental systems

n/a	Involved in the study	
[ ]	[x]	Antibodies
[x]	[ ]	Eukaryotic cell lines
[x]	[ ]	Palaeontology and archaeology
[ ]	[x]	Animals and other organisms
[x]	[ ]	Human research participants
[x]	[ ]	Clinical data
[x]	[ ]	Dual use research of concern

Methods

n/a	Involved in the study	
[x]	[ ]	ChIP-seq
[ ]	[x]	Flow cytometry
[x]	[ ]	MRI-based neuroimaging

Antibodies

Antibodies used	For secretion profiling (bulk and single-cell) antibodies, please refer to Table 1.

	Antibodies for flow cytometry:<br>CD16/32 (clone 93), 1:200, eBioscience (cat#: 14-0161-82)<br>anti-Nos2-AF488 (clone CXNFT), 1:500, eBioscience (cat#: 53-5920-82)<br>anti-Arg1-APC (polyclonal), 1:10, R&D (cat#: IC5868A)<br>anti-pSTAT1(Y701)-AF488 (clone 58D6), 1:50, Cell Signaling Technologies (cat#: 9174S)<br>anti-pSTAT6(Y641)-AF647 (clone D8S9Y), 1:50, Cell Signaling Technologies (cat#: 10205S)
Validation	Antibodies from Cell Signaling Technologies (according to website): "Each antibody has passed rigorous application-specific testing standards, and is routinely test our antibodies on multiple species including human, monkey, mouse, and rat."<br><br>Antibodies from R&D Systems (according to website): "R&D Systems takes rigorous steps towards antibody validation and reproducibility. We have been since the beginning. For 30 years, we have used our industry-leading production standards and quality control specifications to develop antibodies that can be relied on for specificity and reproducibility. By developing and testing our products in-house, we can ensure a validated and specific antibody."<br><br>Antibodies from eBioscience (according to website): "Antibodies are verified by Cell treatment to ensure that the antibody binds to the antigen stated. Target verification by cell treatment can be based on enrichment, depletion or translocation of the protein of interest."

Animals and other organisms

Policy information about studies involving animals; ARRIVE guidelines recommended for reporting animal research

Laboratory animals	Bone marrow was extracted from 6-8 week old male or female C57BL/6J mice.
Wild animals	No wild animals were used in this study.
Field-collected samples	No field-collected samples were used in this study.
Ethics oversight	All animal experiments were performed according to the approved protocols of the Yale University Institutional Animal Care and Use Committee.

Note that full information on the approval of the study protocol must also be provided in the manuscript.

Flow Cytometry

Plots

Confirm that:

- [x] The axis labels state the marker and fluorochrome used (e.g. CD4-FITC).
- [x] The axis scales are clearly visible. Include numbers along axes only for bottom left plot of group (a 'group' is an analysis of identical markers).
- [x] All plots are contour plots with outliers or pseudocolor plots.
- [x] A numerical value for number of cells or percentage (with statistics) is provided.

Methodology

Sample preparation	Macrophages were lifted with gentle scraping in ice-cold PBS + 5 mM EDTA. For intracellular protein staining, cells were blocked with Fc receptor (CD16/CD32) antibody (eBioscience, clone 93) on ice for 15 mins in FACS buffer (PBS + 2% FBS). Then the cells were fixed with Cytofix/Cytoperm (BD Biosciences) and stained in 50 µl with anti-Nos2-AlexaFluor488 at 1:500 dilution (eBioscience, clone CXNFT) and anti-Arg1-APC at 1:10 dilution (R&D, polyclonal) for an hour at 4°C. For phospho-flow, cells were fixed immediately after lifting with PhosFlow Fix buffer for 10 mins at 37ºC, and subsequently permeabilized with PhosFlow Perm Buffer III for thirty minutes on ice (BD Biosciences). Cell suspensions were then blocked with Fc receptor antibody as above, and stained with anti-pSTAT1(Y701)-AlexaFluor488 (Cell Signaling Technologies, clone 58D6) and anti-pSTAT6(Y641)-AlexaFluor647 (Cell Signaling Technologies, clone D8S9Y).
Instrument	All data was acquired on an Accuri B6 flow cytometer (BD Biosciences).
Software	All data was analyzed with FlowJo (FlowJO, LLC).
Cell population abundance	At least 30,000 cells per sample were analyzed for each experiment.
Gating strategy	FSC and SSC were set to remove cell debris, dead cells and doublets. Gating was then set using relevant isotype controls.

- [x] Tick this box to confirm that a figure exemplifying the gating strategy is provided in the Supplementary Information.
